# Comparison of Prognostic Performance Between a Machine Learning Model and Manually Measured Grey-White-Matter Ratio on Early Brain Computed Tomography After Out-of-Hospital Cardiac Arrest

**DOI:** 10.7759/cureus.109468

**Published:** 2026-05-22

**Authors:** Fumiya Inoue, Yohei Ono, Yuji Okazaki, Toshihisa Ichiba

**Affiliations:** 1 Graduate School of Public Health, St. Luke's International University, Tokyo, JPN; 2 Emergency Department, Hiroshima City Hiroshima Citizens Hospital, Hiroshima, JPN

**Keywords:** brain, computed tomography, grey-white matter ratio, machine learning, out-of-hospital cardiac arrest

## Abstract

Objectives

Early prediction of neurological outcomes in patients with out-of-hospital cardiac arrest (OHCA) is critical for guiding treatment decisions. Machine learning (ML) model and grey-white matter ratio (GWR), both derived from brain computed tomography (CT), can be used to predict the neurological outcome. However, their relative performance shortly post-return of spontaneous circulation (ROSC) and whether combining the ML model with prehospital information can improve predictive performance remains unclear. This study aimed (1) to compare the predictive performance for poor neurological outcome between the ML model and manually measured GWR in the early phase after ROSC in patients with OHCA, and (2) to assess the predictive ability of the combination of the ML model and prehospital information.

Methods

This single-center retrospective study included adult patients who underwent brain CT within two hours post-ROSC. The endpoint was consecutive coma post-ROSC. Three slice levels (basal ganglia, centrum semiovale, high convexity) of brain CT images were used to generate the ML model and the GWR. Residual Network 101 (ResNet-101) with transfer learning was constructed in the ML model.

Results

Among the 143 cases, 88 patients had a persistent coma, and 55 awoke from coma. Across 10 repeated five-fold cross-validations, the area under the receiver operating characteristic curves (AUCs) for predicting persistent coma between the three-slice ensembled ML model and the average GWR were not significantly different (ML model: 0.796 (interquartile range (IQR): 0.737-0.826), GWR: 0.821 (IQR: 0.763-0.854); p = 0.121). In the regression analysis, the AUC of the model based on prehospital information was 0.846 (95% CI: 0.772-0.92), which improved to 0.905 (95% CI: 0.856-0.953) after adding the ML score.

Conclusion

The ML model achieved moderate predictive performance, with no significant difference compared with the conventional GWR method. The combination of the ML model and prehospital information could improve predictive performance.

## Introduction

Hypoxic-ischemic brain injury (HIBI) is the leading cause of mortality and morbidity in post-cardiac arrest syndrome (PCAS) [1]. Early prediction of neurological outcomes in patients with PCAS is critical for guiding treatment decisions, including the use of veno-arterial extracorporeal membrane oxygenation (VA-ECMO), implementation of targeted temperature management (TTM), and decisions regarding the discontinuation of resuscitative efforts.

Brain computed tomography (CT) is one of the common modalities for detecting brain injury and predicting poor neurological outcome [2]. The brain edema can be quantified as the grey-white matter ratio (GWR) on brain CT [3]. Although several studies have investigated the GWR on brain CT, its predictive ability within a few hours after return of spontaneous circulation (ROSC) remains limited [4-6]. Since the GWR is typically measured manually, it is subject to inter-observer variability [7]. Several studies have demonstrated strong predictive performance of machine learning (ML), particularly convolutional neural networks (CNNs), in brain CT after cardiac arrest [8-14]. However, few studies using an ML approach have focused on prognostication within the first few hours post-ROSC [9,13]. Moreover, since many of these studies did not adequately assess generalizability through repeated cross-validation, it remains unclear whether an ML approach can consistently outperform the conventional GWR method. In addition, although a multimodal approach rather than reliance on a single finding is recommended for neuroprognostication, few studies have investigated whether integrating ML models with other prognostic factors can improve predictive performance.

This study aimed (1) to evaluate the predictive performance for neurological outcomes of an ML model developed from brain CT acquired within two hours post-ROSC by repeated cross-validation and compare it with that of the conventional GWR method, and (2) to assess the predictive ability of the combination of the ML model with prehospital information.

## Materials and methods

Study design and setting

This single-center, retrospective cohort study included patients who presented to the emergency department of Hiroshima City Hiroshima Citizens Hospital, a Japanese urban tertiary care hospital, between 1 January 2007 and 31 December 2019. All the patients with out-of-hospital cardiac arrest (OHCA) and ROSC were screened for eligibility. Study inclusion criteria included: (1) 20 years old or older, (2) underwent brain CT within two hours post-ROSC, (3) were unable to follow commands after ROSC, and (4) were admitted to the intensive care unit (ICU). Exclusion criteria included: (1) cardiac arrest due to stroke or trauma, (2) severe disability or coma before cardiac arrest, defined as Glasgow-Pittsburgh Cerebral Performance Category (CPC) scores of 3-5, (3) do not attempt resuscitation (DNAR) status at ICU admission, and (4) dead in the emergency department. This study was approved by the Institutional Review Board of Hiroshima City Hiroshima Citizens Hospital (approval number: 2025-2). Given the retrospective nature of the study, the requirement for written informed consent was waived. The study adhered to the Strengthening the Reporting of Observational Studies in Epidemiology statement [15].

Data collection

The following patient data were extracted: age, sex, witnessed cardiac arrest, bystander-initiated cardiopulmonary resuscitation (CPR), initial cardiac rhythm at the scene, time interval from collapse to ROSC, time interval from ROSC to first brain CT, the etiology of cardiac arrest, and management after admission (TTM, ECMO). Shockable cardiac rhythm was defined as the presence of ventricular fibrillation or pulseless ventricular tachycardia. ROSC was defined as at least one minute of continuous confirmation of pulsation. In patients who underwent extracorporeal CPR, the time of ECMO pump activation was considered the time of ROSC. Three levels of brain slices: the basal ganglia (BG), the centrum semiovale (CS), and the high convexity (HC) were extracted for the ML model and the evaluation of GWR values. CT slice selection was conducted by two emergency physicians (FI and TI).

Endpoints

The primary outcome was persistent coma, defined as the inability to follow commands from hospital admission until 28 days post-ROSC or until death. Two emergency physicians (YO and TI) assessed the presence of persistent coma by reviewing medical records. The control group consisted of patients who awoke from a coma. The secondary outcome was CPC 3-5 at 28 days post-ROSC, defined as an unfavorable neurological outcome [16]. In cases of discrepancies regarding the outcomes, the physicians discussed and resolved them. We selected persistent coma as the primary outcome to avoid outcome misclassification associated with the conventional CPC scale. For instance, CPC 5 (death) can include patients who regained consciousness but later died from non-neurological causes. Since these patients do not exhibit HIBI, using CPC-based outcomes could misclassify them and potentially impair the predictive performance of the ML model.

CT scanning and image preprocessing procedures

**Figure 1:**
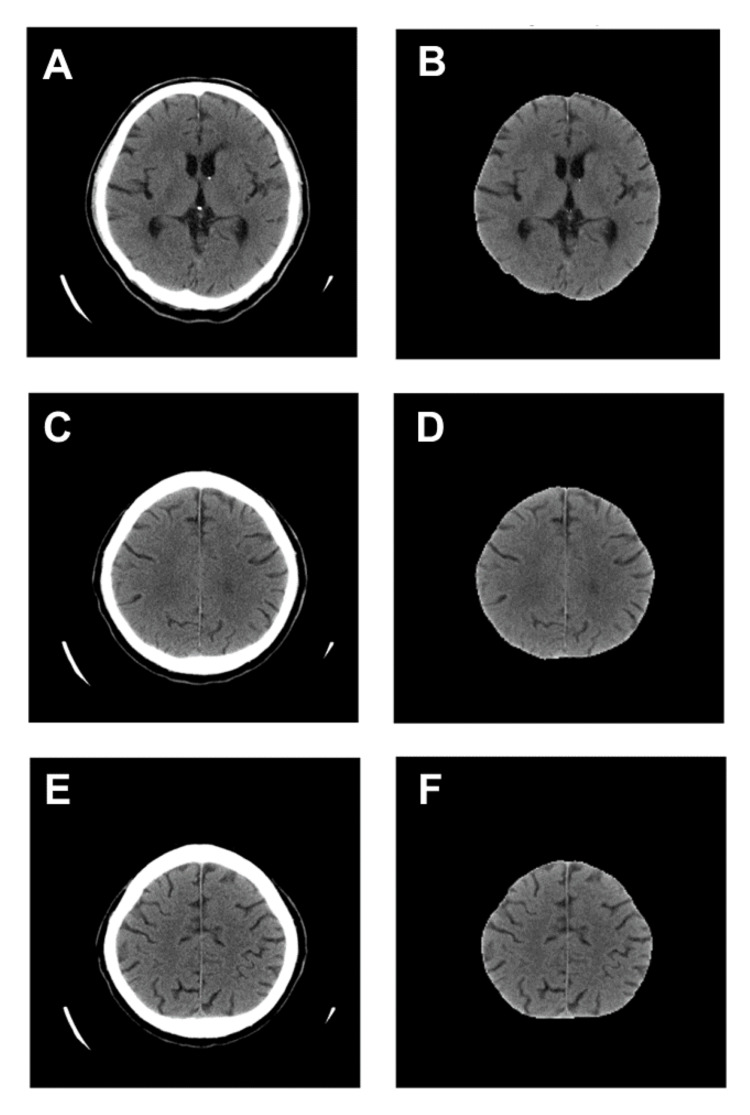
Examples of skull stripping procedure (A) Original image in the basal ganglia slice. (B) Skull-stripped image in the basal ganglia slice. (C) Original image in the centrum semiovale slice. (D) Skull-stripped image in the centrum semiovale slice. (E) Original image in the high convexity slice. (F) Skull-stripped image in the high convexity slice. The figure was created using Python version 3.12 (Python Software Foundation, Wilmington, Delaware, USA).

Non-contrast CT scans were acquired using scanners from multiple vendors: GE Medical Systems (LightSpeed Ultra (n = 65), BrightSpeed (n = 53), HiSpeed (n = 24); GE Healthcare, Chicago, Illinois, USA), and Toshiba Corporation (Asteion (n = 1); Toshiba Medical Systems Corporation, Otawara, Tochigi, Japan). In-plane image dimensions were 512 x 512, with a median slice thickness of 5 mm (interquartile range (IQR): 5-5). The median reconstruction field of view was 250 mm (IQR: 250-250). All scans were acquired with a tube voltage of 120 kV. Digital Imaging and Communications in Medicine (DICOM) CT images were loaded using Python version 3.12 (Python Software Foundation, Wilmington, Delaware, USA) and converted to Hounsfield units (HU) based on the rescale slope and rescale intercept. The CT images were skull-stripped using the following procedure: the brain and cerebrospinal fluid regions were masked using a 0-80 HU window [17,18], and the largest connected component was extracted, with all other regions set to 0 HU. Finally, the 512 × 512-pixel images were resized to 224 × 224 and were normalized to the 0-1 range by dividing pixel values by a fixed constant (255). Examples of the skull-stripped procedure in each slice are shown in Figure 1.

ML model training

A Residual Network 101 (ResNet-101) with transfer learning, initialized with weights pre-trained on ImageNet, was used to train our model [19,20]. We determined that ResNet-101 was the optimal model among ResNet-18, 34, 50, 101, and 152 based on the results of the validation cohort (Table 1).

**Table 1:** Performance of transfer-learning trained models for predicting persistent coma on the validation cohort of the basal-ganglia level Data are presented as median (SD). AUC: area under the receiver operating characteristic curve; SD: standard deviation

Model	Accuracy (%) (SD)	Sensitivity (%) (SD)	Specificity (%) (SD)	Precision (%) (SD)	F1 score (%) (SD)	AUC (SD)	Loss (SD)
ResNet 18	65.2 (11.7)	78.6 (19.7)	55.6 (24.4)	69.2 (13.5)	73.3 (14.2)	0.698 (0.138)	0.658 (0.153)
ResNet 34	65.2 (9.8)	73.3 (18.4)	55.6 (26.5)	72.7 (10.5)	74.1 (10.7)	0.706 (0.135)	0.68 (0.141)
ResNet 50	65.2 (11.2)	78.6 (20.3)	62.5 (26.9)	71.4 (9.9)	75.7 (12)	0.698 (0.145)	0.663 (0.13)
ResNet 101	69.6 (11.2)	78.6 (20.7)	66.7 (24.5)	75 (10.4)	75 (15.1)	0.754 (0.11)	0.625 (0.095)
ResNet 152	65.2 (11)	71.4 (21.6)	62.5 (24.3)	75 (10.9)	74.1 (14.5)	0.73 (0.12)	0.638 (0.085)

**Figure 2:**
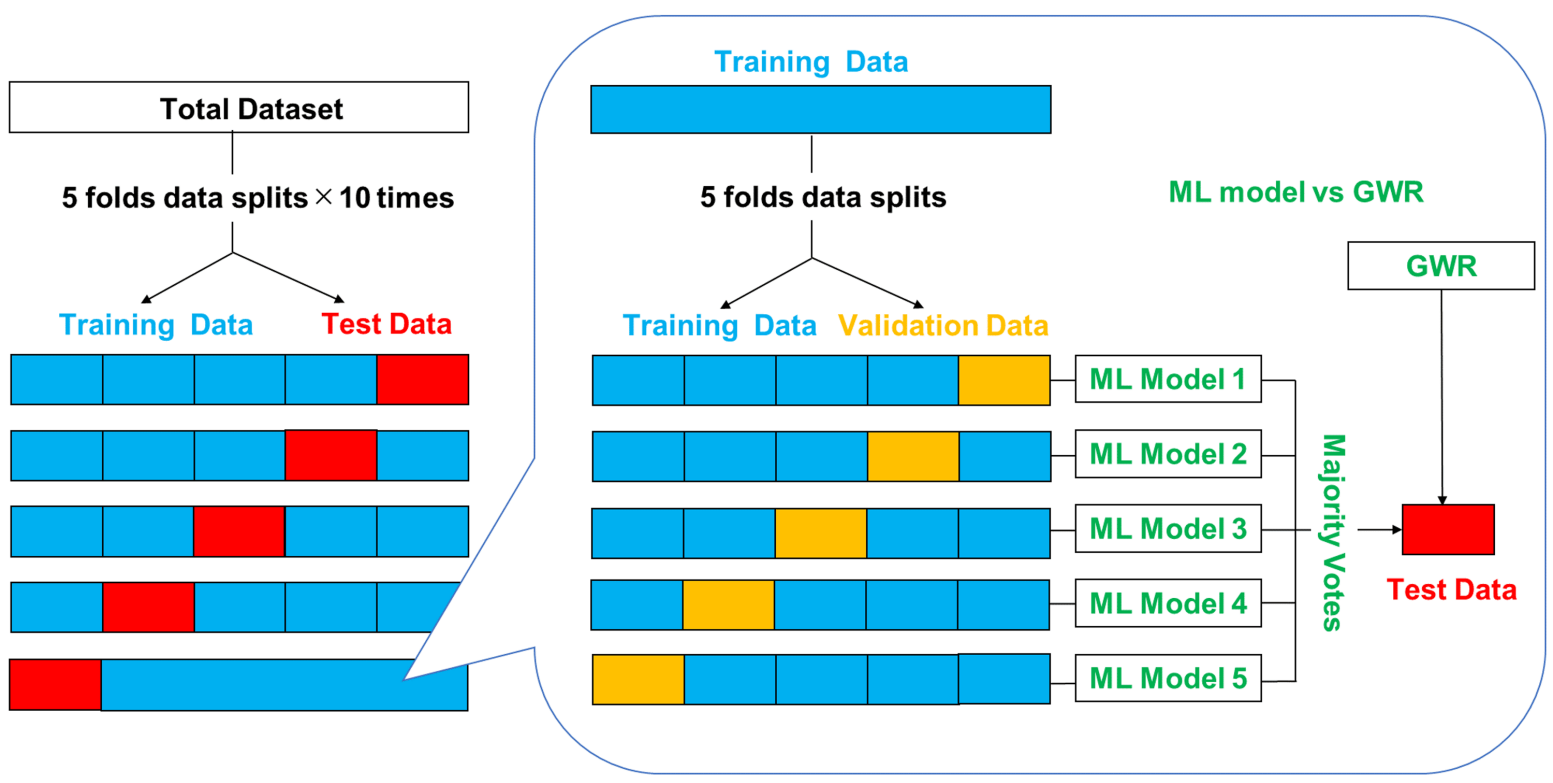
Machine learning (ML) model training and evaluation For each slice, the dataset was divided into five folds, resulting in five pairs of training and test datasets. This process was repeated 10 times randomly, resulting in 50 model tests in total. Each training dataset was further divided into five folds to generate five pairs of training and validation datasets, and five models were trained from each training dataset. Predictions on the test datasets were made by majority voting across the five ML models and compared with predictions based on the grey-white matter ratio (GWR). The figure was created using Microsoft PowerPoint (version 2016; Microsoft Corp., Redmond, WA, USA).

The final fully connected layer was replaced with a new linear layer with two output nodes to perform binary classification (persistent coma vs. awakening from coma). To address class imbalance, we used a class-weighted cross-entropy loss with weights computed from the training set. Input images were augmented during training with random transformations including resized cropping (scale: 0.9-1.0, ratio: 0.95-1.05), rotation (±15°), affine perturbations (translation: ±5%, scale: 0.95-1.05, shear: 5°), horizontal flipping (p=0.5), and brightness jittering (±10%). Models were trained using the Adam optimizer with an initial learning rate of 1 × 10^-4^ [21]. The learning rate was reduced by a factor of 2 whenever the cross-entropy loss on the validation cohort plateaued for two epochs. All trainings were performed with early stopping (maximum number of epochs: 50, minibatch size: 8). The early stopping criteria were defined as when the cross-entropy loss on the validation cohort did not decrease for 10 epochs. Model training and evaluation were conducted using stratified five-fold cross-validation to maintain the class distribution across folds. To prevent data leakage, patients were assigned to one of five folds so that data from a single patient did not appear in more than one training or test set. The five-fold cross-validation was repeated 10 times. For each fold, the training set was further divided into five subsets to train five separate models. Each slice (BG, CS, HC) produced five predictions for each test image, which were aggregated using majority voting (Figure 2).

Finally, predictions from all three slices (15 models in total) were combined using majority voting to generate the final classification for each patient (the three-slice ensembled ML model). The average of votes obtained from 10 times repeated five-fold cross-validation in the three-slice ensembled ML model was defined as the ML score. Gradient-weighted class activation mapping (Grad-CAM) was conducted to visualize model attention [22]. Model training and visualization were conducted using Python version 3.12. The predictive performance of the ML models and the GWR was compared separately for each CT slice level (BG, CS, HC). Furthermore, the three-slice ensembled ML model was compared with the average GWR [23].

GWR value measurements

The GWR values were manually measured by two physicians (FI and TI), with each patient case evaluated by a single physician. CT attenuation was measured in HU by placing an elliptical region of interest (ROI) within axial sections (10-15 mm^2^). At the BG level, HU values were recorded bilaterally at the level of the caudate nucleus (CN), putamen (PU), corpus callosum (CC), and posterior limb of the internal capsule (PLIC). Furthermore, medial cortex and medial white matter were recorded bilaterally at the level of the CS (MC1 and MWM1, respectively) and HC (MC2 and MWM2, respectively). GWR-BG was calculated as (CN + PU)/(CC + PLIC), GWR-CS was calculated as (MC1)/(MWM2), and GWR-HC was calculated as (MC2)/(MWM2). GWR-Cerebrum was calculated as (MC1 + MC2)/(MWM1 + MWM2). We calculated average GWR values as the mean of the GWR-BG and GWR-Cerebrum [23].

Statistical analysis

Patients were categorized into two groups based on persistent coma or awakening from coma. First, we compared the clinical characteristics between the two groups. Second, we compared the area under the receiver operating characteristic curves (AUCs) of the ML model and the GWR using the Wilcoxon signed-rank test for paired data, reporting both the z-score and p-value. Third, we calculated performance metrics for the ML model and the GWR, including accuracy, sensitivity, specificity, precision, and F1 score. The threshold for each GWR was set based on the Youden index derived from the entire cohort. We calculated the predicted probabilities of persistent coma, which were estimated from univariate logistic regression models with either the ML score or the average GWR as a predictor. Fourth, multivariable logistic regression analyses were performed to evaluate the predictive performance of the ML score and the GWR in combination with prehospital information. Initially, a model including only prehospital information (age, sex, witnessed cardiac arrest, initial rhythm, and cardiac arrest time) was constructed (prehospital model) [24,25]. Subsequently, we built models by adding the ML score, the GWR, or both to the prehospital model. For each model, the Akaike information criterion (AIC), Bayesian information criterion (BIC), and AUC were calculated. Complete-case analysis was performed for the multivariable analyses. Finally, to evaluate the impact of training data size on the performance of the ML model, we trained models using 50%, 75%, and 100% of the training dataset and compared their AUCs for predicting persistent coma. All statistical analyses were conducted using the R software package version 4.0.2 (R Foundation for Statistical Computing, Vienna, Austria), the STATA software package version 18.0 (StataCorp LLC, College Station, TX, US), and Python version 3.12. Statistical significance was defined as p<0.05.

## Results

**Figure 3:**
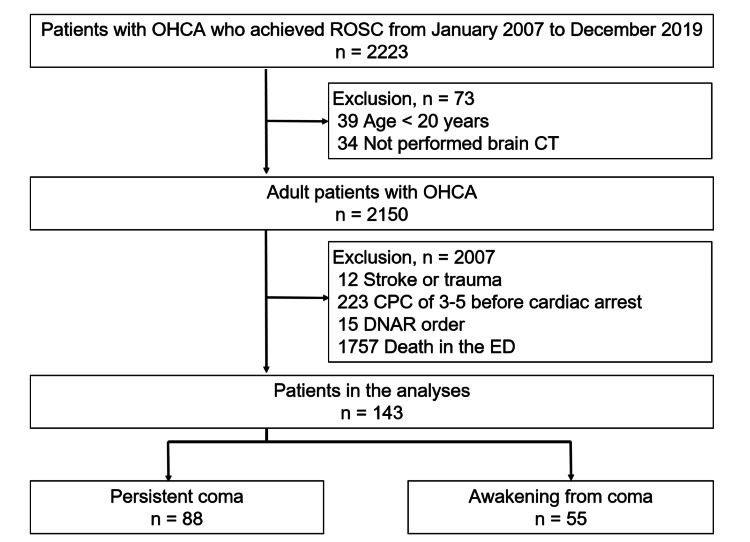
Flow chart of study participant enrollment OHCA: out-of-hospital cardiac arrest; ROSC: return of spontaneous circulation; CT: computed tomography; CPC: cerebral performance category; DNAR: do not attempt resuscitation; ED: emergency department. The figure was created using Microsoft PowerPoint (version 2016; Microsoft Corp., Redmond, WA, USA).

Of the 2,223 OHCA patients who achieved ROSC, 143 patients were included in the analysis (Figure 3).

Among the 143 cases, 88 patients had persistent coma (61.5%), and 55 awoke from coma (38.5%). CPC 3-5 at 28 days was identified in 93 patients (65%), and CPC 1-2 in 50 patients (35%). Among those who awoke from a coma, five patients died from non-neurological causes within 28 days.

Comparison of baseline characteristics according to the presence of persistent coma

Comparisons between the two groups are detailed in Table 2.

**Table 2:** Comparison of baseline characteristics between persistent coma and awakening from coma Data are presented as median (interquartile range) for continuous variables or as N (percentage) for categorical variables. Missing data: Witnessed cardiac arrest = 1, bystander CPR = 1, time interval from collapse to ROSC = 8. CPR: cardiopulmonary resuscitation; ROSC: return of spontaneous circulation; CT: computed tomography; ECMO: extracorporeal membrane oxygenation; CPC: cerebral performance category.

Variables	Total (n=143)	Persistent coma (n=88)	Awakening from coma (n=55)
Age (years)	68 (55-76)	70 (63-79)	61 (47-74)
Male sex, n (%)	97 (67.8)	62 (75.5)	35 (63.6)
Witnessed cardiac arrest, n (%)	116 (81.1)	68 (77.3)	48 (87.3)
Bystander CPR, n (%)	72 (50.3)	34 (38.6)	38 (69.1)
Initial rhythm, n (%)			
Shockable	63 (44.1)	25 (28.4)	38 (69.1)
Non-shockable	80 (55.9)	63 (71.6)	17 (30.9)
Time interval from collapse to ROSC (min)	28 (20-40)	31 (24-41)	20 (10-30)
Time interval from ROSC to first brain CT (min)	22 (13-40)	20 (12-40)	25 (15-43)
Etiology of cardiac arrest			
Cardiac, n (%)	93 (65)	48 (54.5)	45 (81.8)
Non-cardiac, n (%)	50 (35)	40 (45.5)	10 (18.2)
Target temperature management, n (%)	74 (51.7)	43 (48.9)	31 (56.4)
ECMO, n (%)	6 (4.2)	3 (3.4)	3 (5.5)
CPC at 28 days, n (%)			
CPC 1	42 (29.4)	0	42 (76.4)
CPC 2	8 (5.6)	0	8 (14.5)
CPC 3	5 (3.5)	5 (5.7)	0
CPC 4	39 (27.3)	39 (44.3)	0
CPC 5 (coma)	1 (0.7)	1 (1.1)	0
CPC 5 (death)	48 (33.6)	43 (48.9)	5 (9.1)

Across the study population, the median age was 68 years (IQR: 55-76). Of the 143 patients, 97 (67.8%) were males, 116 (81.1%) had witnessed cardiac arrest, 72 (50.3%) received bystander-initiated CPR, and 63 (44.1%) presented with a shockable initial rhythm. The median time interval from collapse to ROSC was 28 min (IQR: 20-40), and the median time interval from ROSC to the first brain CT was 22 min (IQR: 13-40). Female, witnessed cardiac arrest, bystander-initiated CPR, shockable initial rhythm, and cardiac cause of arrest in the persistent coma group were more than 10% less frequent than in the group that awoke from coma.

Comparison of the predictive performances between ML models and the GWR

**Figure 4:**
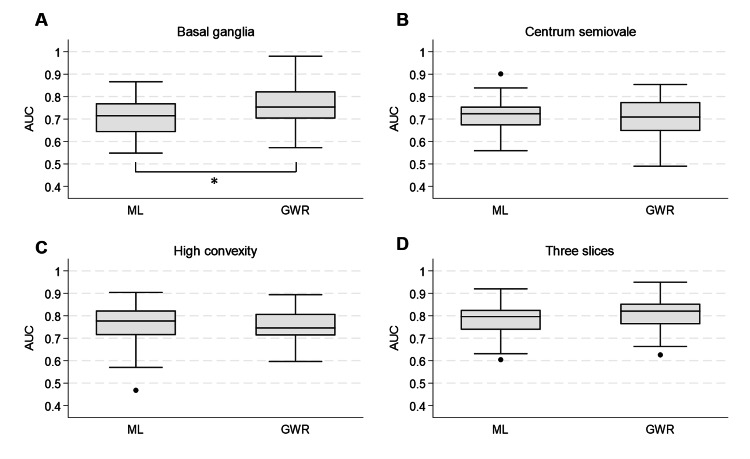
Boxplots comparing the AUC for predicting persistent coma between ML models and the GWR (A) Comparison of AUC in the basal ganglia slice. AUCs in the machine learning (ML) model and grey-white matter ratio (GWR) were 0.715 (interquartile range (IQR): 0.642-0.770) and 0.753 (IQR: 0.702-0.823), respectively. ML showed significantly lower AUC (z=-2.4, p=0.017). (B) Comparison of AUC in the centrum semiovale slice. AUCs in the ML model and the GWR were 0.723 (IQR: 0.672-0.755) and 0.709 (IQR: 0.646-0.775), respectively. AUCs between the ML and the GWR were not significantly different (z=1.11, p=0.271). (C) Comparison of AUC in a highly convexity slice. AUCs in the ML model and the GWR were 0.777 (0.714-0.823) and 0.746 (0.712-0.808), respectively. AUCs between the ML and the GWR were not significantly different (z=0.921, p=0.364). (D) Comparison of AUCs between the three-slice ensembled ML model and the average GWR. AUCs in the three-slice ensembled ML model and the average GWR were 0.796 (0.737-0.826) and 0.821 (0.763-0.854), respectively. AUCs between the ML and the GWR were not significantly different (z=-1.55, p=0.121). The black lines represent median values. Boxes represent IQR. Whiskers cover values within 1.5 IQR of the 25th and 75th percentiles, respectively. Dots are outliers. Asterisks indicate statistically significant differences. AUC: area under the receiver operating characteristic curve The figure was created using the STATA software package version 18.0 (StataCorp LLC, College Station, TX, US).

Comparisons of the AUCs for predicting persistent coma between ML models and the GWR in each slice are detailed in Figure 4.

Among the ML models, the three-slice ensembled ML model achieved the highest AUC. Among the GWRs, the average GWR showed the best AUC. AUCs for predicting persistent coma in the three-slice ensembled ML model and the average GWR were not significantly different (ML model: 0.796 (IQR: 0.737-0.826), GWR: 0.821 (IQR: 0.763-0.854), z=-1.55, p=0.121). Other performance for predicting persistent coma of the ML model and the GWR are detailed in Table 3.

**Table 3:** Performances of the ML model and GWR for predicting persistent coma Data are presented as median (SD). Thresholds for GWR in the basal ganglia, centrum semiovale, high convexity, and average GWR were set at 1.26, 1.21, 1.20, and 1.21, respectively, based on the Youden index calculated from the total cohort. SD: standard deviation; ML: machine learning; GWR: grey-white matter ratio

Model	Accuracy (%) (SD)	Sensitivity (%) (SD)	Specificity (%) (SD)	Precision (%) (SD)	F1 score (%) (SD)
Basal ganglia ML	65.5 (9.5)	64.7 (16.1)	72.7 (16.8)	75 (8.8)	68.8 (12.8)
Centrum semiovale ML	64.3 (7.4)	64.7 (14.4)	68.2 (16.6)	75.6 (8.3)	69.5 (9.2)
High convexity ML	69 (7.9)	65.7 (13.3)	72.7 (17.8)	81.3 (10.6)	71.8 (8.8)
Three-slice ensembled ML	69 (5.9)	66.7 (11.2)	72.7 (12.3)	82.1 (7.4)	72.5 (6.8)
Basal ganglia GWR	75 (8.7)	77.8 (10.3)	72.7 (14.6)	81 (8.2)	78.8 (7.6)
Centrum semiovale GWR	67.9 (7.2)	51.5 (11)	90.9 (6.9)	90.9 (7.7)	65.5 (9.8)
High convexity GWR	66.7 (8)	50 (11.2)	90.9 (6.4)	90.9 (7.6)	64.3 (10.6)
Average GWR	75.9 (7.3)	70.6 (9.7)	81.8 (7.3)	87.5 (6)	78.8 (7.7)

**Figure 5:**
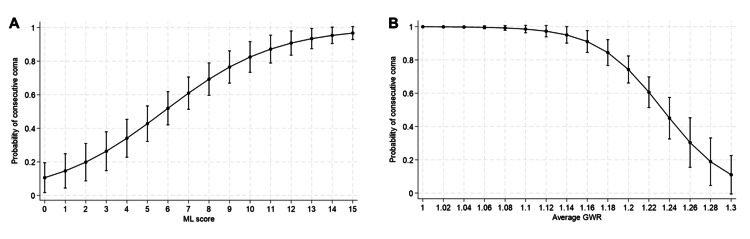
Predicted probability of persistent coma by ML score and average GWR (A) Predicted probability of persistent coma by ML score. (B) Predicted probability of persistent coma by average GWR. Error bars represent 95% confidence intervals. ML: machine learning; GWR: grey-white-matter ratio The figure was created using the STATA software package version 18.0 (StataCorp LLC, College Station, TX, USA).

Among the ML models, the three-slice ensembled ML had the best performances in accuracy (69% (standard deviation (SD): 5.9)), sensitivity (66.7% (SD: 11.2)), specificity (72.7% (SD: 12.3)), precision (82.1% (SD: 7.4)), and F1 score (72.5% (SD: 6.8)). Among all the models, the average GWR had the best accuracy (75.9 (SD: 7.3)), F1 score (78.8 (SD: 7.7)), and Yoden index (sensitivity: 70.6 (SD: 9.7), specificity: 81.8 (SD: 7.3)). The predicted probabilities of persistent coma according to the ML score or the average GWR are shown in Figure 5.

The probability of persistent coma increased with higher ML scores and with lower GWR values.

Predictive performance of the ML model and the GWR combined with prehospital information

Multivariable logistic regression analyses for persistent coma are detailed in Table 4.

**Table 4:** Multivariable logistic regression analysis for predictors of persistent coma * ML score is a continuous variable (ranging from 0 to 15), representing the mean number of votes assigned by the three-slice ensembled ML model. OR: odds ratio; CI: confidence interval; ML: machine learning; GWR: grey-white-matter ratio; NA: not applicable; AIC: Akaike information criterion; BIC: Bayesian information criterion; AUC: area under the receiver operating characteristic curve

Variables	Univariable analysis	Multivariable analysis OR (95% CI)		
	OR (95% CI)	Prehospital	+ ML score	+ GWR
ML score*	1.45 (1.26-1.65)	NA	1.49 (1.24-1.8)	NA
Average GWR (per 0.01)	0.73 (0.64-0.83)	NA	NA	0.72 (0.61-0.85)
Age (years)	1.03 (1.01-1.06)	1.03 (1.01-1.06)	1.03 (1-1.06)	1.06 (1.02-1.1)
Gender (male)	1.36 (0.66-2.79)	1.79 (0.68-4.69)	1.37 (0.43-4.35)	2.06 (0.64-6.67)
Witnessed	0.38 (0.15-1.01)	0.38 (0.11-1.31)	0.26 (0.07-1.03)	0.24 (0.05-1.26)
Initial rhythm (shockable)	0.18 (0.08-0.37)	0.27 (0.11-0.65)	0.18 (0.07-0.55)	0.36 (0.12-1.08)
Cardiac arrest time (min)	1.1 (1.05-1.14)	1.1 (1.06-1.15)	1.1 (1.05-1.15)	1.08 (1.03-1.12)
	AIC	135.6	112.3	116
	BIC	153.1	132.6	136.4
	AUC (95%CI)	0.847 (0.773-0.921)	0.908 (0.861-0.955)	0.892 (0.836-0.948)

Adjusted odds ratios (ORs) of the ML scores in “Prehospital + ML model” were 1.49 (95% confidence interval (CI): 1.24-1.8). Adjusted ORs of the average GWRs in “Prehospital + GWR model” were 0.72 (95% CI: 0.61-0.85). AUCs in the “Prehospital model”, “Prehospital + ML model”, and “Prehospital + GWR model” were 0.847 (95% CI: 0.773-0.921), 0.908 (95% CI: 0.861-0.955), and 0.892 (95% CI: 0.836-0.948). The “Prehospital + ML” model had the lowest AIC (112.3) and BIC (132.6) among all models.

Comparison of AUCs by the size of the training data set

**Figure 6:**
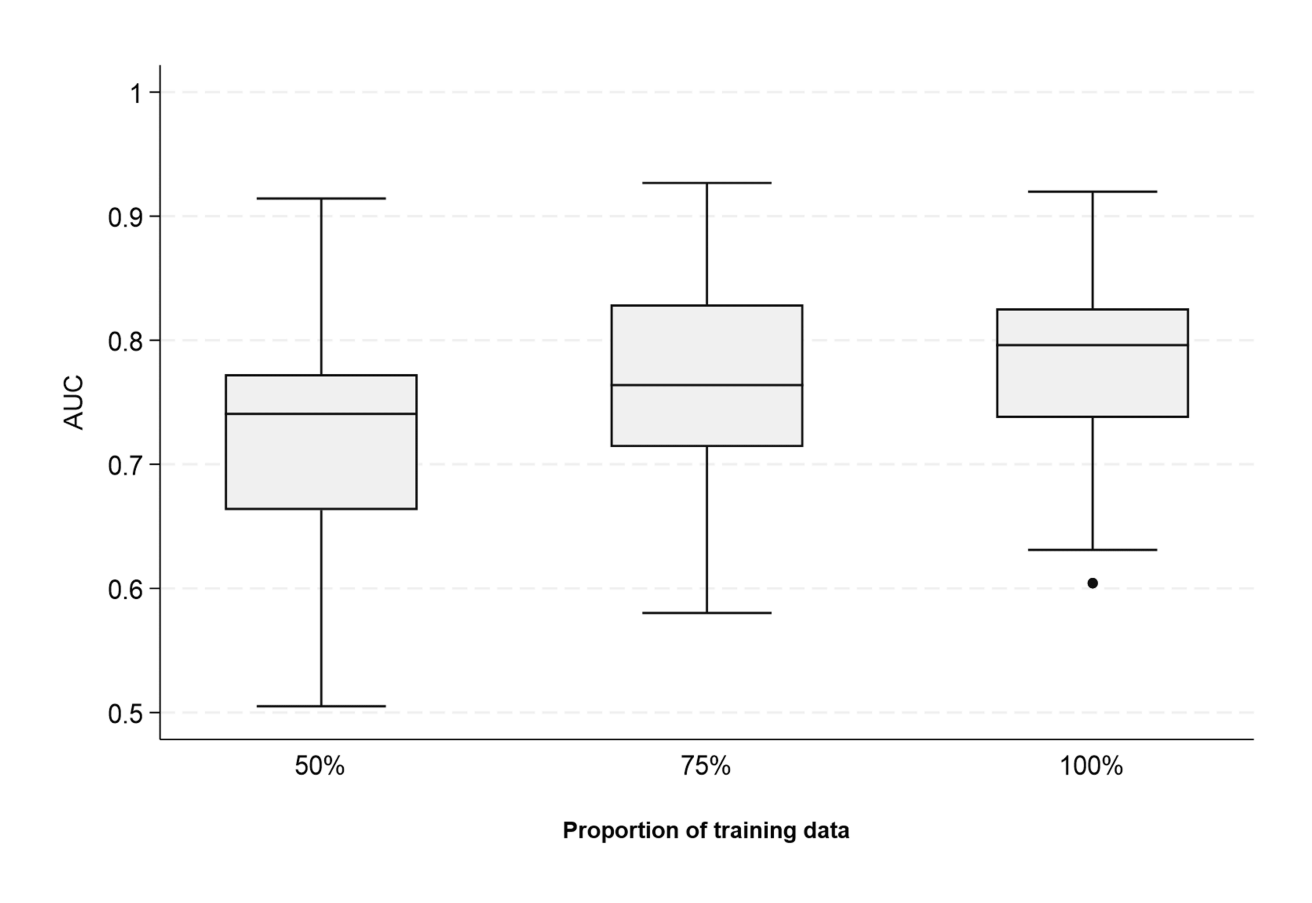
Effect of training dataset size on model performance AUCs for predicting persistent coma in the three-slice ensembled machine learning model with 50%, 75%, and 100% of training data were 0.741 (interquartile range (IQR): 0.663-0.773), 0.764 (0.714-0.829), and 0.796 (0.737-0.826), respectively. AUC: area under the receiver operating characteristic curve The figure was created using the STATA software package version 18.0 (StataCorp LLC, College Station, TX, USA).

AUCs for predicting persistent coma in models trained with 50%, 75%, and 100% of training data were 0.741 (IQR: 0.663-0.773), 0.764 (IQR: 0.714-0.829), and 0.796 (IQR: 0.737-0.826), respectively (Figure 6).

Model performance improved as the size of the training dataset increased.

Model visualization

**Figure 7:**
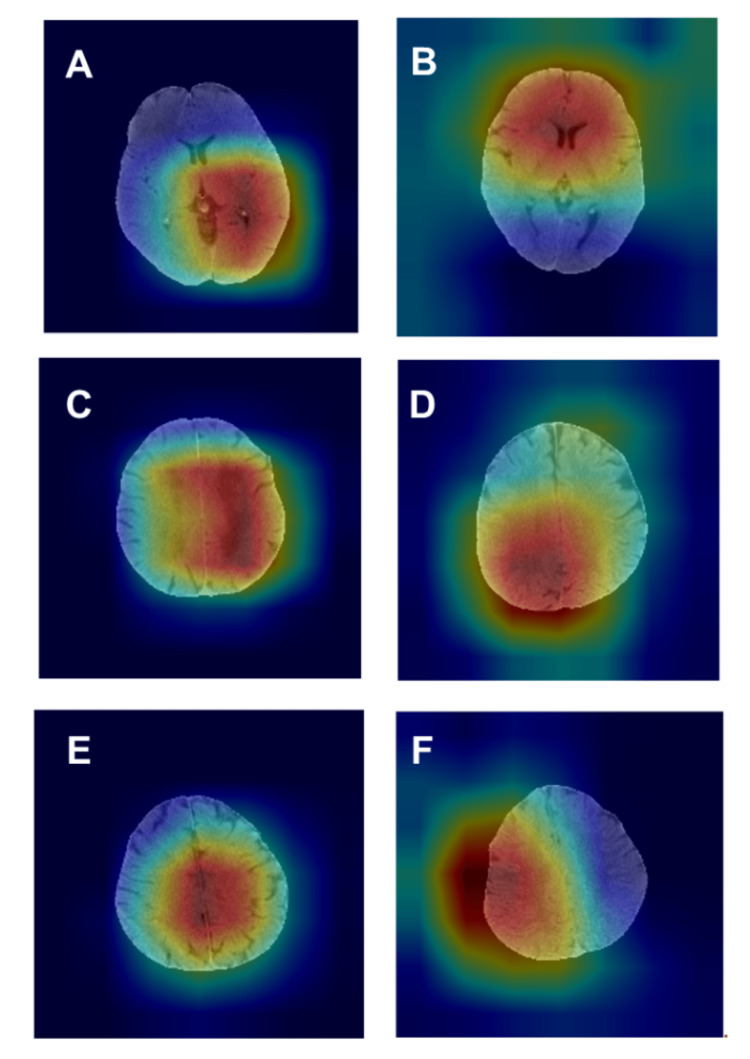
Examples of gradient-weighted class activation mapping (Grad-CAM) for individual computed tomography scans For each slice, one model from a single seed was selected, and the test set case with the highest predicted probability for persistent coma and awakening from coma was chosen for visualization. Attention maps are shown overlaid on a skull-stripped brain image (40% opacity). The area under the curves of the models to which Grad-CAM was applied was 0.778 for the basal ganglia slice, 0.717 for the centrum semiovale slice, and 0.763 for the high convexity slice. (A) The attention map for a basal ganglia slice was correctly classified as persistent coma (persistent coma probability: 100%). (B) The attention map for a basal ganglia slice was correctly classified as awakening from coma (persistent coma probability: 4%). (C) The attention map for a centrum semiovale slice was correctly classified as persistent coma (persistent coma probability: 100%). (D) The attention map for a centrum semiovale slice was correctly classified as awakening from coma (persistent coma probability: 1%). (E) The attention map for a highly convexity slice was correctly classified as persistent coma (persistent coma probability: 100%). (F) The attention map for a highly convexity slice was correctly classified as awakening from coma (persistent coma probability: 7%). The figure was created using Python version 3.12 (Python Software Foundation, Wilmington, Delaware, USA).

Examples of true-positive and true-negative test cases with the highest predicted probabilities for persistent coma and awakening from coma in each slice are shown in Figure 7.

Analyses for the secondary outcome

**Figure 8:**
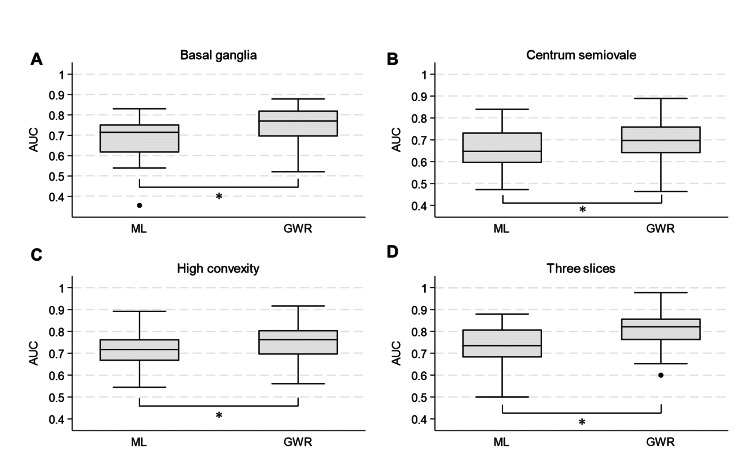
Boxplots comparing the area under the receiver operating characteristic curve for predicting Cerebral Performance Category scores of 3-5 at 28 days between machine learning models and the grey-white matter ratio (A) Comparison of AUC in the basal ganglia slice. AUCs in the ML model and the GWR were 0.714 (interquartile range (IQR): 0.616-0.753) and 0.77 (0.694-0.821), respectively. ML showed significantly lower AUC (z=-3.28, p<0.001). (B) Comparison of AUC in the centrum semiovale slice. AUCs in the ML model and the GWR were 0.647 (0.595-0.733) and 0.697 (0.639-0.761), respectively. ML showed significantly lower AUC (z=-2.15, p=0.031). (C) Comparison of AUC in a highly convexity slice. AUCs in the ML model and the GWR were 0.717 (0.666-0.764) and 0.762 (0.694-0.806), respectively. ML showed significantly lower AUC (z=-2.36, p=0.018). (D) Comparison of AUC in the average model combining three slices. AUCs in three slices ensembled ML model and the GWR were 0.735 (0.682-0.808) and 0.821 (0.761-0.858), respectively. ML showed significantly lower AUC (z=-4.47, p<0.001). The black lines represent median values. Boxes represent IQR. Whiskers cover values within 1.5 IQR of the 25th and 75th percentiles, respectively. Dots are outliers. Asterisks indicate statistically significant differences. AUC: area under the receiver operating characteristic curve; ML: machine learning; GWR: grey-white matter ratio The figure was created using the STATA software package version 18.0 (StataCorp LLC, College Station, TX, USA).

The AUC for predicting CPC 3-5 at 28 days after ROSC in the three-slice ensembled ML model was significantly lower than that in the average GWR (ML model: 0.735 (IQR: 0.682-0.808), GWR: 0.821 (IQR: 0.761-0.858), z=-4.47, p<0.001) (Figure 8).

Other performances for predicting CPC 3-5 at 28 days after ROSC of the ML model and the GWR were detailed in Table 5.

**Table 5:** Performance of the ML model and GWR for predicting Cerebral Performance Category scores of 3-5 at 28 days Data are presented as median (SD). * Thresholds for GWR in the basal ganglia, centrum semiovale, high convexity, and average GWR were set at 1.26, 1.22, 1.2, and 1.21, respectively, based on the Youden index calculated from the total cohort. ML: machine learning; GWR: grey-white matter ratio; SD: standard deviation

Model	Accuracy (%) (SD)	Sensitivity (%) (SD)	Specificity (%) (SD)	Precision (%) (SD)	F1 score (%) (SD)
Basal ganglia ML	64.9 (8.9)	67.5 (17)	60 (21.2)	77.4 (9.8)	72.5 (11)
Centrum semiovale ML	62.1 (9.2)	57.9 (16.2)	65 (18.6)	75 (8.5)	67.6 (9.1)
High convexity ML	67.9 (7.5)	66.7 (14.7)	70 (17.7)	80 (8.7)	72.7 (9.3)
Three slice ensembled ML	67.9 (8.5)	68.4 (14.4)	70 (16.2)	80 (8.1)	73 (9.9)
Basal ganglia GWR*	75 (7.4)	77.8 (9.5)	70 (12.3)	83.8 (6.5)	80 (6.6)
Centrum semiovale GWR*	64.3 (8.4)	51.3 (12.2)	90 (6.1)	92.3 (5.9)	64.4 (11.2)
High convexity GWR*	69 (7.7)	52.6 (11.7)	100 (4)	100 (4.1)	69 (10.2)
Average GWR*	72.4 (7.3)	66.7 (10.2)	90 (7.6)	90.5 (5.6)	76.2 (7.4)

In the multivariable logistic regression analysis, AUCs in “Prehospital model”, “Prehospital + ML model”, and “Prehospital + GWR model” were 0.846 (95% CI: 0.772-0.92), 0.905 (95% CI: 0.856-0.953), and 0.89 (95% CI: 0.833-0.947) (Table 6).

**Table 6:** Multivariable logistic regression analysis for predictors of Cerebral Performance Category scores of 3-5 at 28 days * ML score is a continuous variable (ranging from 0 to 15), representing the mean number of votes assigned by the three-slice ensembled ML model. OR: odds ratio; CI: confidence interval; ML: machine learning; GWR: grey-white matter ratio; NA: not applicable; AIC: Akaike information criterion; BIC: Bayesian information criterion; AUC: area under the receiver operating characteristic curve

Variables	Univariable analysis	Multivariable analysis OR (95% CI)		
	OR (95% CI)	Prehospital	+ ML score	+ GWR
ML score*	1.39 (1.2-1.61)	NA	1.38 (1.15-1.66)	NA
Average GWR (per 0.01)	0.73 (0.64-0.83)	NA	NA	0.69 (0.58-0.83)
Age (years)	1.04 (1.01-1.06)	1.04 (1.01-1.07)	1.03 (1-1.06)	1.07 (1.03-1.11)
Gender (male)	1.31 (0.63-2.71)	1.97 (0.71-5.49)	1.55 (0.44-5.5)	2.42 (0.66-8.8)
Witnessed	0.39 (0.14-1.11)	0.32 (0.09-1.2)	0.22 (0.05-0.99)	0.19 (0.03-1.07)
Initial rhythm (shockable)	0.14 (0.06-0.3)	0.19 (0.07-0.49)	0.17 (0.05-0.52)	0.23 (0.07-0.73)
Cardiac arrest time (min)	1.1 (1.05-1.15)	1.11 (1.06-1.16)	1.11 (1.05-1.16)	1.08 (1.03-1.14)
	AIC	123	111.1	103.5
	BIC	140.4	131.4	123.8
	AUC (95%CI)	0.846 (0.772-0.92)	0.905 (0.856-0.953)	0.89 (0.833-0.947)

## Discussion

This study demonstrated that the AUC of the ML model from brain CT acquired within two hours post-ROSC for predicting persistent coma was not significantly different from that of the manually measured GWR. The combination of the ML model and prehospital information improved the predictive performance.

Few studies have compared the predictive performance of ML models with that of the GWR for the prognosis of OHCA in the early phase post-ROSC. One study showed that the AUC of an ML model based on brain CT images obtained in the immediate post-resuscitation phase for predicting poor neurological outcomes was superior to that of the GWR [9]. However, the internal validity of that study was limited because the model evaluation was based on a single data split without cross-validation. In medical imaging studies with limited sample sizes, models are prone to high variance. Therefore, repeated cross-validation is essential for reliable assessment of predictive performance [26]. Our study showed that the AUCs of the ML model and the GWR were not significantly different with the repeated cross-validation. The limited size of the training dataset may explain why the ML model did not outperform the GWR. However, the AUC in the ML model increased progressively with larger training samples, suggesting that future studies with a larger sample size could enable the ML model to surpass the performance of GWR. For performance metrics other than AUC, the ML model did not outperform GWR. However, in this study, the cutoff value for the GWR was optimized based on the present cohort; therefore, other performance metrics cannot be directly compared in this retrospective analysis. Future prospective studies with a predefined GWR cutoff are warranted for the fair performance comparison of the ML model with the GWR method.

The AUC of the ML model or the GWR alone indicated only moderate predictive performance in our study. One possible explanation for the limited predictive performance of both methods is that HIBI may be difficult to identify on brain CT within two hours post-ROSC. However, the combination of ML score and prehospital information improved predictive performance, suggesting that the ML score provides complementary information to prehospital data. Prehospital information, such as initial rhythm, witnessed status, and arrest duration, allows high specificity and sensitivity in outcome prediction [27]. However, since these prehospital variables are inherently subjective, it is important to combine them with objective information, such as a CT image, for a multimodal assessment [2]. In the acute setting, where rapid decisions regarding interventions, such as TTM and ECMO, are required, a semi-automatically derived ML score may be particularly useful, potentially offering advantages over the manually measured GWR for timely prognostic evaluation.

Manual measurement of the GWR poses the risk of interrater variability, and the standardized automatic assessment may be warranted for clinical practice [7]. Several studies have demonstrated that automated measurement of the GWR can serve as a standardized and reliable predictor of neurological outcomes [28,29]. However, automated measurement of the GWR evaluates only the gray-white matter contrast and cannot assess morphological abnormalities, such as sulcal effacement. In contrast, CNNs have the potential to comprehensively extract and evaluate features from medical images, including both contrast and morphological characteristics. However, it is difficult for CNNs to explain how and what they have learned to perform a classification task [30]. Although GradCAM was applied to visualize model attention in our study, it was not possible to determine whether the model made judgments based on brain contrast or shape [22]. This may be because the classification task in our study was more complex than simple lesion detection, and the limited sample size might have hindered the development of a sufficiently interpretable model. Since our ML model used ensemble prediction to ensure reproducible and stable predictions, visualization of the entire model was not possible. Further studies comparing ML models trained on sufficiently large datasets with the GWR method are warranted.

This study has several limitations. First, this was a single-center retrospective study, which restricts the generalizability of the results. In addition, the ML model was not validated using an external dataset. However, robust internal validation was performed using 10 times-repeated five-fold cross-validation to assess model stability. Future multicenter studies with external validation are warranted to confirm the generalizability and clinical applicability of our findings. Second, the selection of slices for ML training and GWR measurement was performed by emergency physicians manually. Therefore, the proposed ML model is semi-automated, not fully automated. Fully automated slice selection would require atlas-based registration, which is technically challenging and vulnerable to imaging artifacts [28,29]. Although manual slice selection can be performed quickly without substantial burden in clinical practice, concerns regarding reproducibility remain. Future studies with larger datasets should explore slice-independent approaches, such as three-dimensional CNN models, which may enable fully automated analysis without the need for manual slice selection. Third, our primary outcome was persistent coma until 28 days post-ROSC or until death, which differs from the conventional neurological outcome classification of CPC 1-2 versus CPC 3-5. However, CPC 5 could include patients who regained consciousness but subsequently died from non-neurological causes. Such patients may not have exhibited features of HIBI. Therefore, the use of CPC-based outcomes could lead to misclassification in the context of predicting HIBI, potentially impairing the performance of the ML model. In fact, the ML model trained with the secondary outcome demonstrated a significantly lower AUC than the GWR. Finally, as each patient's GWR was measured by only one of the two physicians, inter-observer variability could not be assessed. Additionally, utilizing the entire cohort to determine GWR thresholds carries a potential risk of optimization bias. This issue might lead to an overestimation of the model’s performance.

## Conclusions

This study demonstrated that the AUCs for predicting persistent coma using the ML model and the manually measured GWR, derived from brain CT acquired within two hours post-ROSC, were not significantly different in repeated cross-validation. Although the predictive performance of the ML score was only moderate, the combination with prehospital information may further improve neuroprognostication. In acute settings requiring rapid decisions, this semi-automated ML model could support timely and standardized prognostic evaluation compared with manual GWR measurements. Larger prospective multicenter studies are warranted to evaluate the ML model performance for neuroprognostication.
